# MALDI-TOF MS: A Quick Method to Detect the Susceptibility of *Fusarium* spp. Clinical Isolates to Amphotericin B

**DOI:** 10.3390/microorganisms11071834

**Published:** 2023-07-18

**Authors:** Patrícia Helena Grizante Barião, Yasna Cayún, Marcela Sepúlveda, Ludmilla Tonani, Otavio Guilherme Gonçalves de Almeida, Pablo Cornejo, Nathalia Dias, Cledir Santos, Marcia Regina von Zeska Kress

**Affiliations:** 1 Departamento de Análises Clínicas, Toxicológicas e Bromatológicas, Faculdade de Ciências Farmacêuticas de Ribeirão, Ribeirão Preto 14040-903, SP, Brazil; 2 Programa de Doctorado en Ciencias Mención Biología Celular y Molecular Aplicada, Universidad de La Frontera, Temuco 4811-230, Chile; 3 Department of Chemical Science and Natural Resources, Universidad de La Frontera, Temuco 4811-230, Chile; 4 Programa de Doctorado en Ciencias de Recursos Naturales, Universidad de La Frontera, Temuco 4811-230, Chile; 5 Escuela de Agronomía, Facultad de Ciencias Agronómicas y de los Alimentos, Pontificia Universidad Católica de Valparaíso, Quillota 2260-000, Chile; 6 Scientific and Technological Bioresource Nucleus (BIOREN), Universidad de La Frontera, Temuco 4811-230, Chile

**Keywords:** MPCC, MIC, fungal resistance

## Abstract

Disseminated fusariosis is treated with amphotericin B and voriconazole. To determine adequate therapy, the minimal inhibitory concentration (MIC) is used. However, MIC analysis is based on visual observation and requires a long period of fungal incubation. The measure of the minimal profile change concentration (MPCC) using MALDI-TOF MS is a quick spectral method that has presented good results in determining the antimicrobial resistance of yeasts. However, there is a lack of information on filamentous fungi. In the present work, 13 *Fusarium* spp. clinical isolates and two reference strains were used. MIC was obtained according to the M38-A2 protocol of the Clinical Laboratory Standards Institute, while MPPC was obtained following the initial steps of the M38-A2 protocol. Both Biotyper and the Rstudio environment were used to analyze mass spectra. For some fungal strains, the data obtained from the software MALDI Biotyper Compass 4.1 led to fuzzy heatmaps resulting in difficult interpretation, while heatmaps obtained using Rstudio tools generated better MPCC resolutions. Herein, 86.6% of the AMB MPCC values were highly correlated with the gold-standard AMB MIC. MALDI-TOF MS is a prominent tool used to determine MPCCs quicker, cost-effectively, and more accurately for *Fusarium* spp. strains. However, better statistical analyses could help measure the technique’s limit detection.

## 1. Introduction

The *Fusarium* genus is composed of fungal species that are ubiquitously distributed in soils, and the genus is associated with other fungal species, such as insect symbionts and plant parasites, which may lead to economic losses in agriculture [1]. In addition, some species in the *Fusarium* genus are also identified as opportunistic pathogens in immunosuppressed patients with fungemia in the clinical context of invasive fusariosis. At the same time, the infections tend to be local in immunocompetent patients, emphasizing onychomycosis and keratitis [2,3]. Due to their ability to infect humans, animals, and plants, *Fusarium* species are considered trans-kingdom pathogens [4,5].

From a taxonomic point of view, *Fusarium* is an enigmatic fungal genus since it is composed of cryptic species that present high phenotypic and genetic similarities, impairing taxonomic resolution and species identification when using routine culturing and biochemical assays [1,6]. The resolution of *Fusarium* species is hard to obtain using traditional identification methods. In clinical routines, these species are grouped in "species complexes", a term used to refer to fungal taxa or lineages with closely related taxonomy and difficulties in species identification [7,8]. About 74 *Fusarium* species related to human pathogenicity are reported in the literature [9]. Among them, the most frequent are *F. solani*, *F. oxysporum*, *F. fujikuroi*, *F. dimerum*, *F. equiseti*, and *F. chlamydosporum* [10,11,12].

Due to the increase in immunocompromised patients, the cases of fusariosis have also dramatically increased in recent decades, especially among neutropenic individuals [13,14,15]. In addition, there is also an increase in antifungal resistance [16,17,18]. Studies developed in the last decade show that *Fusarium* spp. have a high rate of intrinsic resistance to a wide spectrum of antifungal agents that are often used in the medical field, such as azoles, echinocandins, and polyenes [3,19,20,21,22,23]. Furthermore, diverse antifungal susceptibility patterns have been noticed for different species within a single species complex [24,25,26], and high mortality rates due to fusariosis have been observed among immunocompromised patients due to intrinsic resistance to antifungals [9,14,27,28,29,30,31]. Consequently, the World Health Organization (WHO) has recently listed *Fusarium* spp. among the highly antifungal-resistant species. This fungal prioritization list is intended to guide research, development, and public health actions [32].

To determine the in vitro susceptibility of clinical fungal isolates relative to antifungals, the broth microdilution methods based on either the M38-A2 or the E.DEF 9.3.2 protocols are the gold-standard methods. The M38-A2 protocol was launched by the Clinical and Laboratory Standards Institute (CLSI) [33], while the European Committee for Antimicrobial Susceptibility Testing (EUCAST) recommends the E.DEF 9.3.2 protocol [34].

Both protocol M38-A2 and protocol E.DEF 9.3.2 determine the antifungal minimal inhibitory concentrations (MICs) and are considered reproducible for *Fusarium* spp. However, no antifungal breakpoint has yet been established for *Fusarium* spp., as mentioned earlier. This lack of data is due to the gap in clinical trials and knowledge about the mechanisms that trigger resistance among *Fusarium* species [35].

In 2016, Espinel-Ingroff et al. (2016) determined the epidemiological cutoff values (ECVs) as an alternative to evaluating the susceptibility profile of *Fusarium* spp. to antifungals. Therefore, ECV aids in the differentiation between wild-type and non-wild-type strains concerning antifungal susceptibility. Strains with MIC values equal to or above the ECV are considered non-wild-type strains and are possibly resistant to the treatment [35].

The matrix-assisted laser desorption ionization–time of flight mass spectrometry (MALDI-TOF-MS) is a cost-effective analytical method for the rapid phenotypic identification of fungal species. Recently, Gómez-Velázquez et al. (2021) reviewed the application of MALDI-TOF MS on filamentous fungi identification in a clinical mycology laboratory [36]. The fungal identification by MALDI-TOF MS is mainly based on ribosomal protein analysis using minimal sample preparation [37,38].

In addition to ribosomal proteins, other biomarkers (e.g., sugars, carbohydrates, long-chain polymer chitin, and non-ribosomal peptides) of molecular mass ranging between 2000 and 20,000 Da are also important and considered for fungal identification. The fungal biomarkers’ mass spectra are generated as a cellular fingerprint. For fungus identification, only the presence or absence of such peaks (e.g., ribosomal proteins) is considered. In contrast, peak intensities (ions abundance) are irrelevant for fungal identification [36,37].

The minimum profile change concentration (MPCC) is a fast antifungal susceptibility mass-spectrometry-based test. For MPCC analysis, the MALDI-TOF-MS technique is used [39,40,41]. The MPCC analysis follows the same conditions of the MALDI-TOF MS for fungal identification; however, instead of a culture medium, the fungal cells are prepared following the same protocol for MIC analysis [41].

MPCC is the minimal antifungal concentration at which changes are detected in the MALDI-TOF MS spectra of a given strain. Thus, the MPCC can detect proteomic changes in the cell of a given fungus that occur after exposure to antimicrobials and is highly correlated to the MIC. It can formally be defined, according to De Carolis et al. (2012), as “a value defined as the lowest drug concentration at which a spectrum is more similar to the one observed at the maximum concentration than the spectrum observed at the null concentration” [40].

The MPCC method can exclude subjective readings by visualizing variations in the protein composition of microorganisms by comparing mass spectra. The MPCC results are obtained faster than the MIC gold-standard method. The MPCC method has previously been used in studies that assessed the susceptibility of clinically relevant fungi [39,40,41,42,43,44,45].

Marinach et al. (2009) described one of the first preliminary studies using MPCC with fluconazole against *Candida albicans*. According to the authors, the method was accurate, reliable, and fully agreed with the results obtained from the CLSI method. In addition, the MPCC method has been applied to examine the susceptibility of other *Candida* species to different antifungals [41,43,46].

To the best of our knowledge, no study has been conducted thus far on the MPCC method applied to *Fusarium* clinical strains and amphotericin B. Thus, the present study aims to evaluate the feasibility of using MPCC via MALDI-TOF MS as a rapid method to determine the resistance of *Fusarium* spp. to amphotericin B as a way of contributing to the better management and treatment of the infections caused by clinically related *Fusarium* spp. strains.

## 2. Materials and Methods

### 2.1. Clinical Isolates and Strains

Thirteen *Fusarium* spp. clinical isolates and two reference strains (ATCC36031 *F. oxysporum* and ATCC48112 *F. keratoplasticum*) were used in this study. The clinical isolates were obtained from different body sites of human patients from different São Paulo State (Brazil) regions. The fungal dataset comprised *Fusarium oxysporum* (n = 3), *F. keratoplasticum* (n = 5), *F. proliferatum* (n = 2), *F. sacchari* (n = 1), *F. falciforme* (n = 2), *F. petroliphilum* (n = 1), and *F. delphinoides* (n = 1) [47].

### 2.2. Broth Microdilution Susceptibility Method and the Minimal Inhibitory Concentration

Antifungal susceptibility tests were performed according to the broth microdilution susceptibility method using the M38-A2 protocol of the Clinical Laboratory Standards Institute-CLSI [34]. Briefly, fungal isolates were grown on a Potato Dextrose Agar (PDA, 3 g/L potato extract, 20 g/L Glucose, 15 g/L Agar) for conidia production at 28 °C for 3 to 5 days.

A suspension of fungal conidia on distilled sterile water was prepared, filtered through a sterile miracloth filter, and adjusted to a final concentration of 0.4 × 10^4^ to 5 × 10^4^ conidia/mL using a hemocytometer. Both conidia suspensions of 100 µL and 100 µL of serial AMB dilution (0.06 to 32 µg/mL) on an RPMI 1640 culture medium buffered with 0.165 M 3-(N-morpholino) propanesulfonic acid (MOPS), pH 7.0, were transferred into a 96-well microplate, which was incubated at 35 ± 2 °C for 48 h. Both AMB and RPMI 1640 were purchased from Sigma-Aldrich Chemical Corporation.

Data were recorded by visual observation, and the minimal inhibitory concentration (MIC) was defined as the lowest concentration of AMB that produces 100% inhibition with respect to fungal growth. *Aspergillus flavus* (ATCC204304) was used as quality control for the M38-A2 protocol assays.

### 2.3. MALDI-TOF MS Spectra Acquisition

The MPCC is the minimal drug concentration at which the changes in MALDI-TOF MS spectra are detected. The MPPC analysis was performed following the initial steps of the M38-A2 protocol. Serial dilutions of AMB in RPMI medium were prepared in a 96-well microplate. Fungi were inoculated and incubated at 37 °C for 15 h, with shaking at 100 rpm according to the methodology previously established by De Carolis et al. (2012) with modifications [40]. Samples without AMB were used as the control.

For protein extraction, the samples were centrifuged at 13,000 rpm for 2 min, the supernatant was removed, and 200 µL of sterile water was added. The sample was vortexed for 1 min and centrifuged for 2 min at 13,000 rpm. Water (100 µL) and ethanol (300 µL) were added to the resulting mixture. The sample was vortexed for 1 min and centrifuged once again for 2 min at 13,000 rpm.

An aqueous formic acid solution (70% *v*/*v*; 30 µL) was added to the centrifuged sample, which was vortexed for 1 min and centrifuged at 13,000 rpm for 2 min. The resulting supernatant was used for spectrum acquisition. Each sample (1 µL) was deposited in triplicate on a stainless-steel MALDI sample plate.

After drying, 1 µL of alpha-cyano-4-hydroxycinnamic acid matrix solution (CHCA, Fluka, Buchs, Switzerland) saturated in a solution composed of 30% (*v*/*v*) acetonitrile, 69.9% (*v*/*v*) H_2_O, and 0.1% (*v*/*v*) trifluoroacetic acid was gently mixed in each sample on a stainless-steel MALDI sample plate.

**Figure 1:**
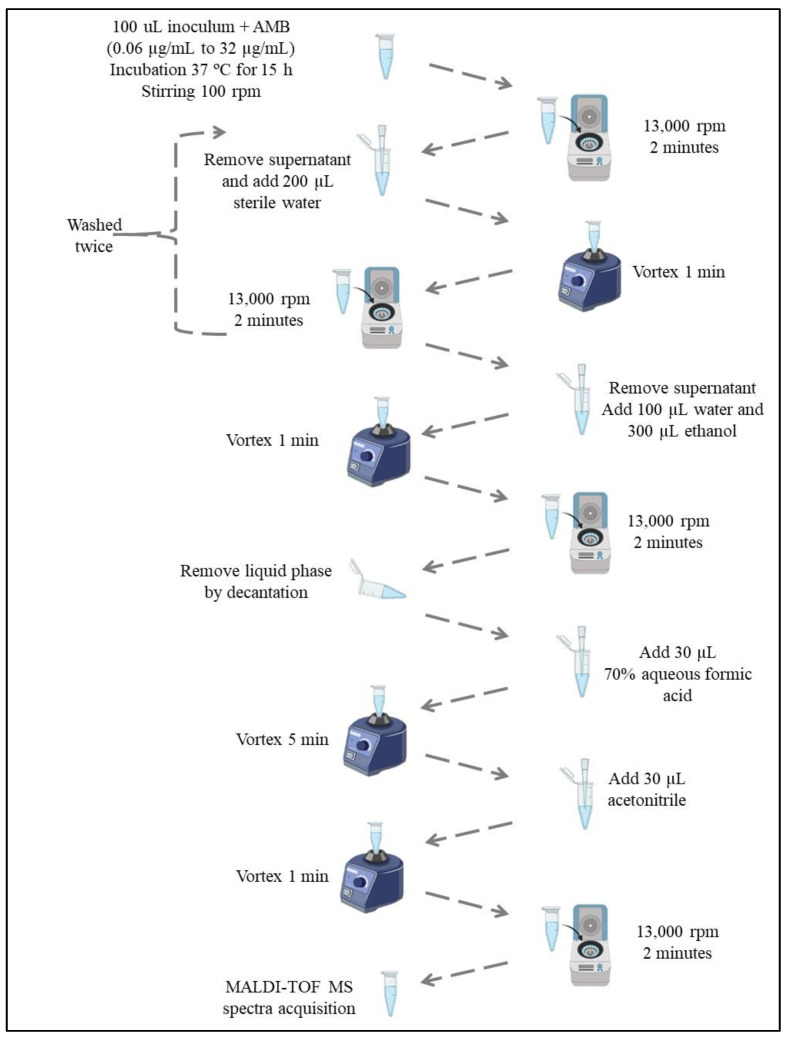
Protein extraction procedure of *Fusarium* spp. clinical isolates for MPCC determination using MALDI-TOF MS.

Afterward, air-dried mass spectra were acquired using the linear and positive modes of MALDI-TOF MS Autoflex Speed (Bruker Daltonics, Bremen, Germany), which was equipped with a smart beam laser source (355 nm) [47] (Figure 1).

Each spectrum was collected as an average of 1200 laser shots with enough energy to produce good spectra without saturation in the range of *m*/*z* from 2000 to 20,000 Da. Before analysis, the equipment was externally calibrated using protein calibration standard I (Bruker Daltonics, Bremen, Germany), which contains insulin, ubiquitin, cytochrome C, and myoglobin.

### 2.4. Data Analysis and MPCC Determination

MPCC determination was carried out using both MALDI Biotyper Compass 4.1 (Bruker Daltonics, Bremen, Germany) and the Rstudio environment [45]. In this latter case, dedicated packages were used: MALDIquantForeign for raw spectra importing and MALDIquant for chemometric analysis. The *m*/*z* range of 3000 to 12,000 Da was used.

For MALDI Biotyper, composite correlation index (CCI) matrices were obtained with the MALDI-TOF MS raw spectra data using Biotyper tools. For Rstudio packages, Pearson correlation index (PCI) matrices were obtained with the raw spectra data in the Rstudio environment using dedicated packages MALDIquantForeign for raw spectra importing and MALDIquant for chemometrics analysis. In this latter case, PCI was calculated using a feature table generated from the processed raw data of each spectrum using the “cor” function, a native function for correlation analysis in the R environment.

For MALDI Biotyper Compass 4.1 statistical software, CCIs were translated into a heatmap using Biotyper tools. For the Rstudio environment package, the heatmaps were plotted using the ggplo2 R package.

The analysis based on both Biotyper and Rstudio packages using the MALDI-TOF MS raw spectra data of *Fusarium* strains incubated with null and serial AMB dilutions (0.06 to 32 µg/mL) resulted in CCI and PCI values, respectively. The results range from 0 to 1, in which the values near zero indicate a low spectral correlation (blue to green in the Biotyper heatmap and light orange to yellow in the Pearson heatmap), while values near 1 represent a high spectral correlation (dark orange to red in Biotyper heatmap and red to dark orange in Pearson heatmap).

In both Biotyper and Rstudio tools, MPCC values were determined for each strain at the minimum concentration of AMB in which the result of the correlation index with the spectrum at the maximum concentration (32 μg/mL AMB) is higher than the result of the correlation index with the control spectra (0 μg/mL AMB).

## 3. Results

For the gold-standard method (M38-A2 protocol), 14 out of 15 *Fusarium* spp. strains (93.3% n = 14/15) showed the AMB MICs ranged from 1 to 4 µg/mL. Clinical isolate LMC7108.01 (*F. keratoplasticum*; 6.7% n = 1/15) presented an AMB MIC of >32 µg/mL. The AMB MIC for the quality control strain (*Aspergillus flavus* ATCC204304) was within the accepted limits of the M38-A2 protocol.

For MPCC analysis, fungus spectra were analyzed after 15 h of fungal incubation upon AMB absence (control) and in AMB concentrations ranging from 0.06 to 32 µg/mL. An AMB MPCC ranging from 1 to 8 µg/mL was observed in 80% (n = 12/15) of *Fusarium* spp. Strains. In addition, 6.7% (n = 1/15) of strains presented an AMB MPCC of >32 µg/mL (LMC7108.01), and 13.3% (n = 2/15) showed an MPCC value of <0.06 µg/mL (LMC7178.01 and LMC7163.01) (Table 1).

**Figure 2:**
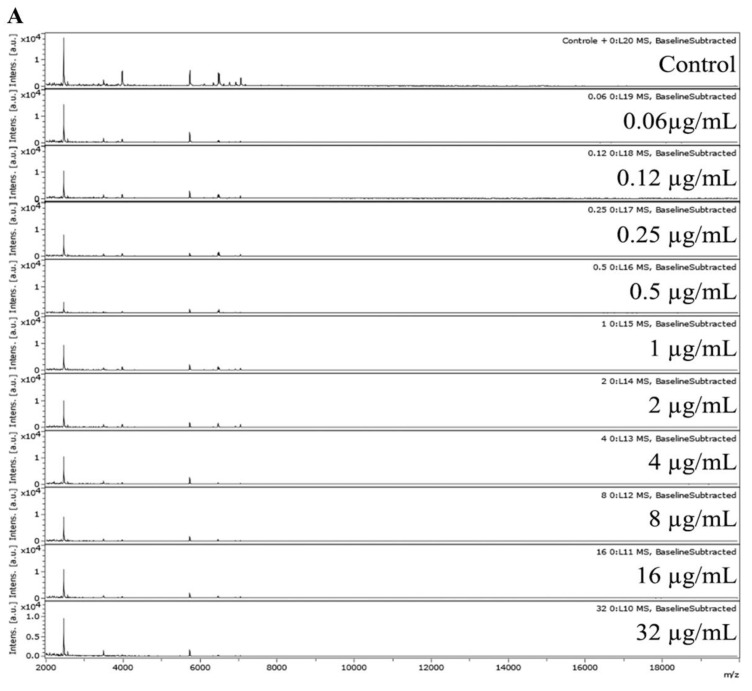

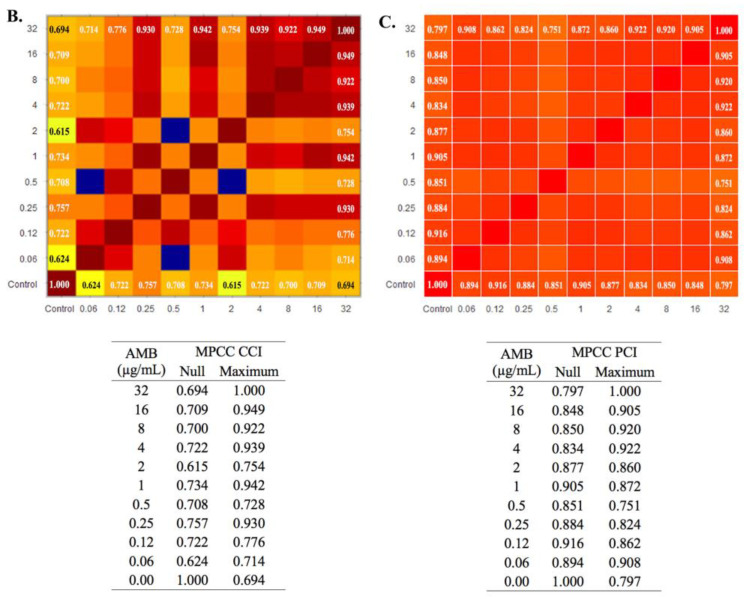
(**A**) MALDI-TOF MS spectra for MPCC the determination of LMC7108.01 *F. keratoplasticum*; (**B**) Biotyper (near zero: blue to green; near 1: dark orange to red) and (**C**) Pearson heatmaps (near zero: yellow to light orange; near 1: dark orange to red). Tables: Biotyper CCI and Pearson PCI. CCI, composite correlation index; PCI, Pearson correlation index.

**Figure 3:**
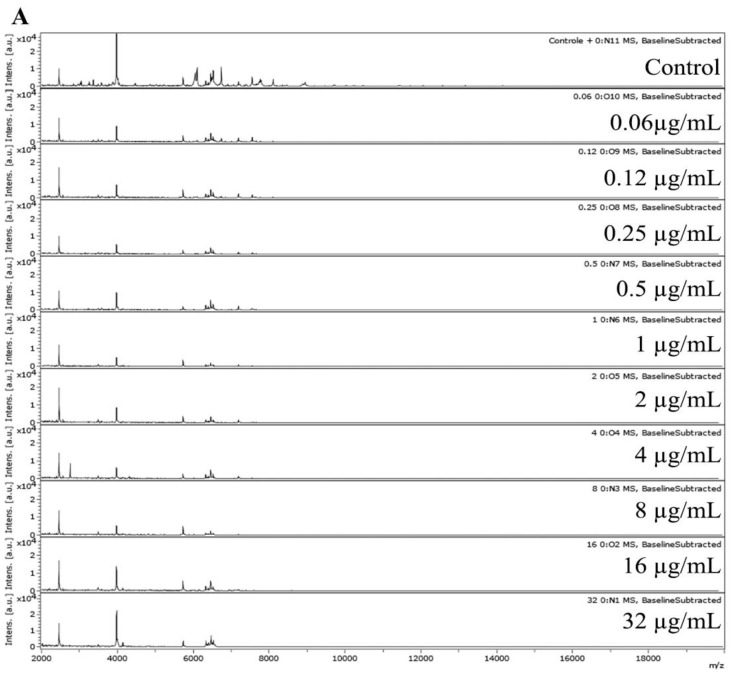

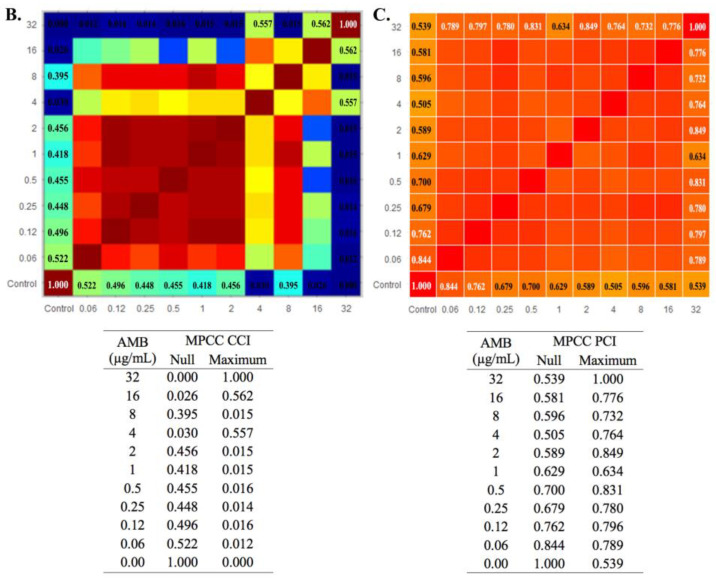
(**A**) MALDI-TOF MS spectra for the MPCC determination of LMC7178.01 *F. falciforme*; (**B**) Biotyper (near zero: blue to green; near 1: dark orange to red) and (**C**) Pearson heatmaps (near zero: yellow to light orange; near 1: dark orange to red). Tables: Biotyper CCI and Pearson PCI. CCI, composite correlation index; PCI, Pearson correlation index.

The MPCC interpretation was based on the similarity of the spectra (correlation index near 1.000) between the maximum AMB concentration (32 µg/mL) and the control (fungus without AMB) or other AMB concentrations (0.06 to 16 µg/mL). Thus, the minimal AMB concentration in which the spectrum was similar to the maximum concentration determines the AMB MPCC. In high MPCC concentration, in the case of >32 µg/mL AMB, the heatmap and correlation indexes show a tendency of values to be near 1.000 relative to all conditions, including the control (null) and >32 µg/mL AMB (maximum) (Figure 2). On the other hand, in low MPCC concentrations (<0.06 µg/mL) and/or undefined MPCC, the heatmap and correlation indexes show a tendency of values to be near 1.000 relative to all conditions, except for the control (null), which presented a low to zero correlation index with the maximum condition (Figure 3).

The MPCC and MIC values were in agreement or exhibited a 1-fold or 2-fold dilution difference for 86.7% (n = 13/15) of *Fusarium* strains (Table 1). The differing AMB MIC and MPCC values occurred for LMC7178.01 and LMC7163.01, representing 13.3% (n = 2/15) of the strains. It is worth noting that, even with up to a 2-fold dilution difference between AMB MPCC and MIC results, all but one strain showed a wild-type profile for AMB. These values follow the epidemiological cutoff values (ECVs) for *Fusarium* spp. [35].

**Figure 4:**
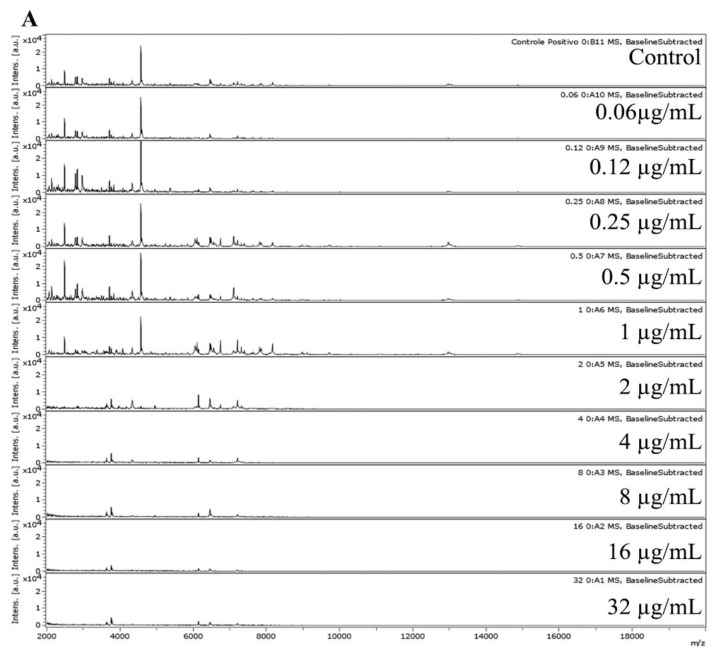

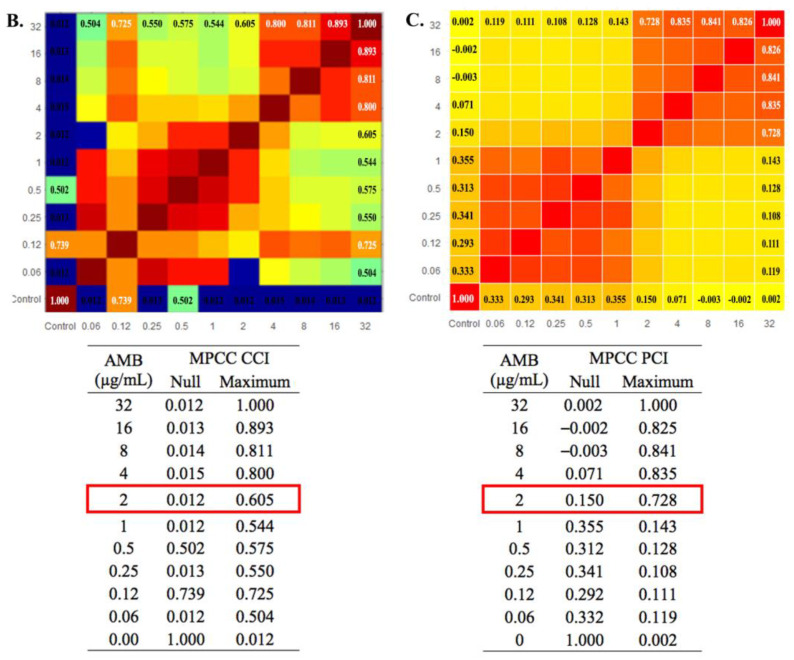
(**A**) MALDI–TOF MS spectra for the MPCC determination of LMC7170.01 *F. oxysporum*; (**B**) Biotyper (near zero: blue to green; near 1: dark orange to red); and (**C**) Pearson heatmaps (near zero: yellow to light orange; near 1: dark orange to red). Tables: Biotyper CCI and Pearson PCI. CCI, composite correlation index; PCI, Pearson correlation index. Red rectangle, AMB MPCC.

Figure 4A–C show an example of MALDI-TOF MS spectra, Biotyper heatmap/CCI matrix, and Pearson heatmap/PCI matrix for the LMC7170.01 (*F. oxysporum*) strain.

Overall, the MALDI-TOF MS spectra were recorded in the range from 2000 to 20,000 Da, where the main biomarkers used for fungus identification appear. Regarding the spectral data for LMC7170.01 *F. oxysporum*, no spectral change is observed from the control (AMB free) to the 0.12 µg/mL AMB spectrum. A light change in the peak intensity of ions is observed from 0.12 to 0.25 µg/mL AMB, where peaks become higher regarding their intensities. Finally, a breakpoint is observed at 2 µg/mL AMB, where at this concentration, changes are clearly observed in the mass spectra (Figure 4A). Despite the observed difference that determines the AMB MPCC for LMC7170.01, MALDI-TOF MS spectra were not entirely easy to interpret using simple spectra visualization.

In the LMC7170.01 sampling, three different spectral patterns (zero to 0.12, 0.25 to 1, and 2 to 32 µg/mL of AMB) were observed, which presented differences in the abundance of some ion peaks (Figure 4A). The difficulty in the protein’s spectral data interpretation is possibly due to the concentration-dependent fungistatic and fungicidal effect of AMB.

The results observed in the LMC7170.01 heatmaps generated by both Biotyper and Rstudio tools agreed with the visual difference observed in LMC7170.01 spectral data (Figure 4B,C). Similar results were observed for the other strains independent of MIC values (Figure 2 and Figure 3). However, although Biotyper heatmaps were often easy to interpret, some were difficult to interpret. Thus, additional analysis was required, and Pearson’s statistical analysis was adopted to analyze all data. The MPCC values established by the Pearson heatmap presented better resolutions for all cases. In addition, breakpoints in CCI and PCI matrices helped establish the MPCC values for both Biotyper and Pearson analyses (Figure 4).

**Figure 5:**
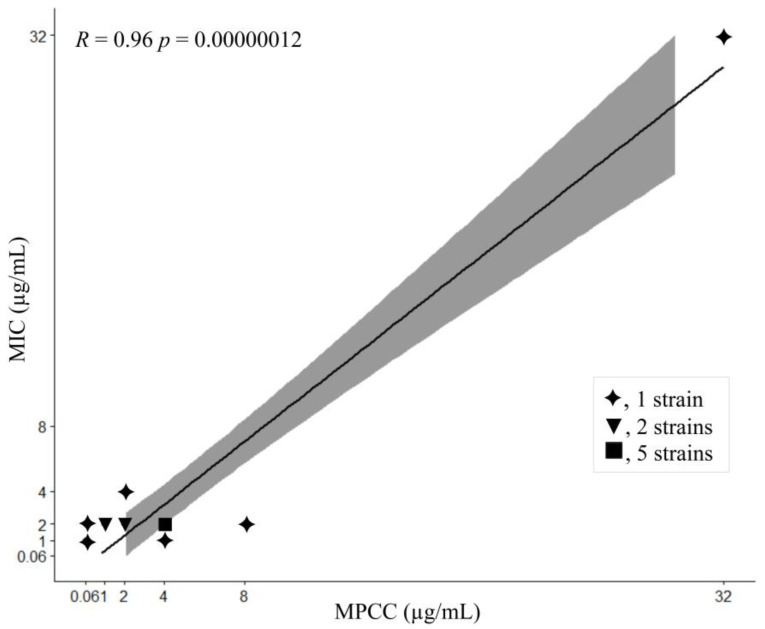
Linear correlation between the AMB MIC and MPCC of 15 *Fusarium* spp. strains.

The correlation of AMB MPCC and MIC values for 15 strains was examined (Table 1 and Figure 4). A significant linear regression correlation (*p* < 0.00000012) and a regression coefficient of 0.96 pointed out a linear association between AMB MPCC and MIC (Figure 5).

## 4. Discussion

An addressed fungal resistance concern is the intrinsic resistance of *Fusarium* spp. relative to a broad spectrum of antifungals that are routinely used in the medical field, such as azoles, echinocandins, and polyenes [3,19,21,22,23,46]. Therefore, the antifungal susceptibility test (AFST) is mandatory in order to understand fungal susceptibility and to improve patient management and treatment.

Broth microdilution methods are the gold-standard protocol for detecting antifungal susceptibility. However, these methods are time-consuming and based on visual determination, which can result in inaccurate information. Innovative methods are currently under development to improve AFST. The MPCC performed by MALDI-TOF MS is considered a promising, reproducible, low-cost, and fast technique that is capable of providing reliable results as it can reduce the analysis times compared to the gold-standard methods used in the routine [48,49].

Different studies describe the application of the MPCC method and its correlation with conventional MIC values for *Candida* spp. [39,40,41,43,46]. Recently, an MPCC protocol was described to determine the antifungal susceptibility of *Aspergillus fumigatus* strains to azoles [48]. These studies have previously been assessed to establish an MPCC method for echinocandins [40,41,44] and triazoles [39,42,43,44,45].

Here, we aimed to establish a MALDI-TOF-MS (MPCC) protocol to determine the antifungal susceptibility of *Fusarium* spp. strains to AMB. Additionally, we correlated it with the MICs obtained using the gold-standard CLSI method. The findings presented herein showed that 86.6% (n = 13/15) of the AMB MPCC values were highly correlated with the gold-standard AMB MIC. These results agree with previous results for *Aspergillus fumigatus*, in which the correlations of MIC and MPCC values were 88.2% and 82.3% for voriconazole and itraconazole, respectively [49].

For *Candida tropicalis*, fluconazole MPCCs were equivalent or one-fold dilution different than the respective fluconazole MIC [45]. In addition, previous studies carried out using the strains of the *Candida parapsilosis* species complex submitted to antifungals micafungin, caspofungin, and anidulafungin reported high concordance with respect to CLSI and MALDI-TOF MS methods [41].

Marinach et al. (2009) showed a maximum acceptable difference of 2-fold dilutions in the correlation between MPCC and MIC values [39]. In the present study, MIC and MPCC values presented comparable AMB concentration ranges and a linear association. Herein, it was possible to directly infer the susceptibility to AMB in terms of MPCC profiles. However, not all heatmaps showed clear results (Figure 2 and Figure 3).

The heatmaps for two clinical isolates (LMC7108.01 and LMC7178.01) were difficult to interpret (Figure 2 and Figure 3). AMB has a fungistatic effect in a concentration-dependent manner, and the fungi can undergo several changes in their protein composition [50]. For some fungal strains, it can generate fuzzy heatmaps that are difficult to interpret. The heatmap obtained using Rstudio tools generated better resolutions relative to MPCC visualization than the data delivered by the MALDI Biotyper Compass 4.1 (Figure 2 and Figure 3).

Herein, we show MALDI-TOF mass spectrometry (MPCC) as a prominent tool to determine the AFST of *Fusarium* spp. strains. It is a faster, cost-effective, and more accurate method compared to the gold-standard method (MIC). However, the statistical package used in MALDI Biotyper Compass 4.1needs to be improved to generate a better image resolution of the heatmaps used in tests involving AMB and the *Fusarium* species related to this work.

In addition, further analyses with more strains of *Fusarium* spp. isolated from different sources and geographic regions could help measure the technique’s limit detection. The great sensitivity of MALDI-TOF MS as an adequate statistical tool could detect minor proteomics changes that can clearly delimit the cutoff for MPCC estimations.

## 5. Conclusions

The minimal profile change concentration (MPCC) is a novel proposal for replacing the labor-intensive minimal inhibitory concentration (MIC) methodology for the rapid AFST screening of clinical fungi strains. MPCC aids the proper stewardship of antifungals rationally. In addition, it correlates well with MICs, justifying its application in routine testing.

Here is the first study in light of AMB MPCC for *Fusarium* spp. clinical strains. MALDI Biotyper Compass 4.1 data generated fuzzy heatmaps with difficult interpretation for some fungal strains. The heatmaps obtained using Rstudio tools (023.03.0-daily+82.pro2) generated better MPCC resolutions once the programming-based analysis overcame the Blackbox problem observed in commercial software using custom analysis designs.

Most *Fusarium* spp. Clinical strains (86.6%) showed AMB MPCC values that are highly correlated with the gold-standard AMB MIC. Thus, MALDI-TOF MS is a prominent tool for determining MPCCs faster, cost-effectively, and more accurately with respect to *Fusarium* spp. strains. However, future studies must delimitate the sensibility of the method and the external factors related to antifungal properties (fungicide or fungistatic effects); sample preparation; inoculum concentration; experimental design variations; and even post-analytic procedures, such as bioinformatics approaches for raw data processing and statistical analysis choice, which taken together influence MPCC determination and visualization.

## Figures and Tables

**Table 1:** Amphotericin B minimal inhibitory concentration (MIC and CLSI broth microdilution) and minimal profile change concentration (MPCC: Biotyper and Rstudio) against *Fusarium* spp. strains.

Species Complex	Species	Strain	MIC (µg/mL)	MPCC (µg/mL)
FOSC	*F. oxysporum*	ATCC48112	2	4
		LMC7137.01	2	4
		LMC7170.01	2	2
FSSC	*F. keratoplasticum*	ATCC36031	2	4
		LMC7108.01	>32 *	>32 *
		LMC7113.02	2	1
		LMC7163.01	1	<0.06
		LMC7205.01	4	2
	*F. falciforme*	LMC7178.01	2	<0.06
		LMC7193.01	2	8
	*F. petroliphilum*	LMC7162.02	2	4
FFSC	*F. proliferatum*	LMC7138.01	2	1
		LMC21300.01	2	4
	*F. sacchari*	LMC21303.01	2	2
FDSC	*F. delphinoides*	LMC7215.01	1	4

FSSC, *F. solani* species complex; FOSC, *F. oxysporum* species complex; FFSC, *F. fujikuroi* species complex; FDSC, *F. dimerum* species complex; * MIC above the epidemiological cutoff value (ECV) comprising ≥97.5% of the statistically modelled population [35].

## References

[1] Lombard L., Sandoval-Denis M., Cai L., Crous P.W. (2019). Changing the game: Resolving systematic issues in key *Fusarium* species complexes. Persoonia.

[2] Lainhart W. (2017). *Fusarium* spp., a Genus of Common Plant Pathogens That Can Cause Devastating, Opportunistic Human Disease. Clin. Microbiol. Newsl..

[3] Herkert P.F., Al-Hatmi A.M.S., de Oliveira Salvador G.L., Muro M.D., Pinheiro R.L., Nucci M., Queiroz-Telles F., De Hoog G.S., Meis J.F. (2019). Molecular characterization and antifungal susceptibility of clinical *Fusarium* species from Brazil. Front. Microbiol..

[4] Gauthier G., Keller N. (2013). Crossover fungal pathogens: The biology andpathogenesis of fungi capable of crossing kingdoms to infect plants and humans. Fung. Genet. Biol..

[5] Van Diepeningen A.D., De Hoog G.S. (2016). Challenges in *Fusarium*, a Trans-Kingdom Pathogen. Mycopathologia.

[6] Summerell B.A. (2019). Resolving *Fusarium*: Current status of the genus. Annu. Rev. Phytopathol..

[7] Kwon-Chung K.J., Bennett J.E., Wickes B.L., Meyer W., Cuomo C.A., Wollenburg K.R., Bicanic T.A., Castañeda E., Chang Y.C., Chen J. (2017). The case for adopting the “species complex” nomenclature for the etiologic agents of cryptococcosis. mSphere.

[8] Geiser D.M., Al-Hatmi A., Aoki T., Arie T., Balmas V., Barnes I., Bergstrom G.C., Bhattacharyya M.K.K., Blomquist C.L., Bowden R. (2021). Phylogenomic analysis of a 55.1 kb 19-gene dataset resolves a monophyletic *Fusarium* that includes the *Fusarium solani* species complex. Phytopathology.

[9] Al-Hatmi A.M., Ende A.G.V.D., Stielow J.B., Van Diepeningen A.D., Seifert K.A., McCormick W., Assabgui R., Gräfenhan T., De Hoog G.S., Levesque C.A. (2016). Evaluation of two novel barcodes for species recognition of opportunistic pathogens in *Fusarium*. Fung. Biol..

[10] Batista M., Costa S., Shikanai-Yasuda M., Moss R. (2013). Current treatment options for invasive aspergillosis. Drugs Today.

[11] Triest D., Stubbe D., De Cremer K., Piérard D., Normand A.-C., Piarroux R., Detandt M., Hendrickx M. (2015). Use of matrix-assisted laser desorption ionization-time of flight mass spectrometry for identification of molds of the *Fusarium* genus. J. Clin. Microbiol..

[12] Zhu Z.X., Zheng L., Hsiang T., Yang G.L., Zhao D.L., Lv B., Chen Y.F., Huang J.B. (2016). Detection and quantification of *Fusarium commune* in host tissue and infested soil using real-time PCR. Plant. Pathology.

[13] Tortorano A.M., Richardson M., Roilides E., van Diepeningen A., Caira M., Munoz P., Johnson E., Meletiadis J., Pana Z.-D., Lackner M. (2014). ESCMID and ECMM joint guidelines on diagnosis and management of hyalohyphomycosis: *Fusarium* spp., *Scedosporium* spp. and others. Clin. Microbiol. Infect..

[14] Harpaz R., Dahl R., Dooling K. (2016). The prevalence of immunocompromised adults: United States, 2013. Open Forum Infect. Dis..

[15] Lass-Florl C., Cuenca-Estrella M. (2017). Changes in the epidemiological landscape of invasive mould infections and disease. J. Antimicrob. Chemother..

[16] Smith K.D., Achan B., Hullsiek K.H., McDonald T.R., Okagaki L.H., Alhadab A.A., Akampurira A., Rhein J.R., Meya D.B., Boulware D.R. (2015). Increased antifungal drug resistance in clinical isolates of *Cryptococcus neoformans* in Uganda. Antimicrob. Agents Chemother..

[17] McCarthy M.W., Kontoyiannis D.P., Cornely O.A., Perfect J.R., Walsh T.J. (2017). Novel Agents and Drug Targets to Meet the Challenges of Resistant Fungi. J. Infect. Dis..

[18] Sikora A., Hashmi M.F., Zahra F. (2023). Candida Auris. StatPearls [Internet].

[19] Lucas J.A., Hawkins N.J., Fraaije B.A. (2015). The evolution of fungicide resistance. Adv. Appl. Microbiol..

[20] Al-Hatmi A.M.S., Meis J.F., De Hoog G.S. (2016). *Fusarium*: Molecular diversity and intrinsic drug resistance. PLoS Pathog..

[21] Ray S., Das S., Suar M., Arora G., Sajid A., Kalia V.C. (2017). Molecular Mechanism of Drug Resistance. Drug Resistance in Bacteria, Fungi, Malaria, and Cancer.

[22] Lagudah E.S., Krattinger S.G. (2019). A new player contributing to durable Fusarium resistance. Nat. Genet..

[23] Blaize M., Normand A.-C., Imbert S., Al-Hatmi A.M.S., Chryssanthou E., Cassaing S., Schuttler C., Hasseine L., Mahinc C., Costa D. (2021). Antifungal susceptibility of 182 *Fusarium* species isolates from 20 European centers: Comparison between EUCAST and gradient concentration strip methods. Antimicrob. Agents Chemother..

[24] O’Donnell K., Sutton D.A., Fothergill A., McCarthy D., Rinaldi M.G., Brandt M.E., Zhang N., Geiser D.M. (2008). Molecular phylogenetic diversity, multilocus haplotype nomenclature, and in vitro antifungal resistance within the *Fusarium solani* species complex. J. Clin. Microbiol..

[25] Al-Hatmi A.M.S., Van Diepeningen A.D., Curfs-Breuker I., De Hoog G.S., Meis J.F. (2015). Specific antifungal susceptibility profiles of opportunists in the *Fusarium fujikuroi* complex. J. Antimicrob. Chemother..

[26] Song Y., Liu X., Yang Z., Meng X., Xue R., Yu J., Al-Hatmi A.M., de Hoog G.S., Li R. (2021). Molecular and MALDI-TOF MS differentiation and antifungal susceptibility of prevalent clinical *Fusarium* species in China. Mycoses.

[27] Anaissie E., Nelson P., Beremand M., Kontoyiannis D., Rinaldi M. (1992). *Fusarium* caused hyalohyphomycosis. An: Overview. Curr. Top. Med. Mycol..

[28] Sutton D.A., Brandt M.E. (2011). Fusarium and other opportunistic hyaline fungi. Manual of Clinical Microbiology.

[29] Moretti M.L., Busso-Lopes A., Moraes R., Muraosa Y., Mikami Y., Trabasso P., Tominaga K., Reichert-Lima F., Lyra L., Gonoi T. (2014). Environment as a potential source of *Fusarium* spp. invasive infections in immunocompromised patients. Open Forum Infect. Dis..

[30] Garnica M., Da Cunha M.O., Portugal R., Maiolino A., Colombo A.L., Nucci M. (2015). Risk factors for invasive fusariosis in patients with acute myeloid leukemia and in hematopoietic cell transplant recipients. Clin. Infect. Dis..

[31] Rotjanapan P., Chen Y.C., Chakrabarti A., Li R.Y., Rudramurthy S., Yu J., Kung H.C., Watcharananan S., Tan A.L., Saffari S.E. (2018). Epidemiology and clinical characteristics of invasive mould infections: A multicenter, retrospective analysis in five Asian countries. Med. Mycol..

[32] WHO (2022). Fungal Priority Pathogens List to Guide Research, Development and Public Health Action.

[33] (2008). Reference Methods for Broth Dilution Antifungal Susceptibility Testing of Filamentous Fungi.

[34] EUCAST (2020). The European Committee on Antimicrobial Susceptibility Testing: Method for the determination of broth dilution minimum inhibitory concentrations of antifungal agents for conidia forming moulds. EUCAST Antifungal MIC Method for Moulds.

[35] Espinel-Ingroff A., Colombo A.L., Cordoba S., Dufresne P.J., Fuller J., Ghannoum M., Gonzalez G.M., Guarro J., Kidd S.E., Meis J.F. (2016). An international evaluation of MIC distributions and ECV definition for *Fusarium* species identified by molecular methods for the CLSI broth microdilution method. Antimicrob. Agents Chemother..

[36] Gómez-Velásquez J.C., Mojica-Figueroa I.L., Santos C., Lima N., Mesa-Arango A.C. (2021). MALDI-TOF MS: Foundations and a practical approach to the clinical relevant filamentous fungi identification. Curr. Fungal Infect. Rep..

[37] Pereira L., Dias N., Santos C., Lima N. (2014). The use of MALDI-TOF ICMS as an alternative tool for Trichophyton rubrum identification and typing. Enfermedades Infecc. Microbiol. Clín..

[38] Rabello V.B.d.S., Corrêa-Moreira D., Santos C., Pinto T.C.A., Procopio-Azevedo A.C., Boechat J., Coelho R.A., Almeida-Paes R., Costa G., Lima N. (2023). Preservation Methods in Isolates of Sporothrix Characterized by Polyphasic Approach. J. Fungi.

[39] Marinach C., Alanio A., Palous M., Fekkar A., Brossas J.-Y., Brun S., Snounou G., Hennequin C., Sanglard D., Datry A. (2009). MALDI-TOF MS-based drug susceptibility testing of pathogens: The example of *Candida albicans* and fluconazole. Proteomics.

[40] De Carolis E., Vella A., Florio A.R., Posteraro P., Perlin D.S., Sanguinetti M., Posteraro B. (2012). Use of matrix-assisted laser desorption ionization-time of flight mass spectrometry (MALDITOF MS) for caspofungin susceptibility testing of *Candida* and *Aspergillus* species. J. Clin. Microbiol..

[41] Roberto A.E.M., Xavier D.E., Vidal E.E., Vidal C.F.d.L., Neves R.P., de Lima-Neto R.G. (2020). Rapid Detection of Echinocandins Resistance by MALDI-TOF MS in *Candida parapsilosis* Complex. Microorganisms.

[42] Vella A., De Carolis E., Vaccaro L., Posteraro P., Perlin D.S., Kostrzewa M., Posteraro B., Sanguinetti M. (2013). Rapid antifungal susceptibility testing by Matrix-Assisted Laser Desorption Ionization Time-of-Flight Mass Spectrometry analysis. J. Clin. Microbiol..

[43] Saracli M.A., Fothergill A.W., Sutton D.A., Wiederhold N.P. (2015). Detection of triazole resistance among *Candida* species by matrix-assisted laser desorption/ionization-time of flight mass spectrometry (MALDI-TOF MS). Med. Mycol..

[44] Vella A., De Carolis E., Mello E., Perlin D.S., Sanglard D., Sanguinetti M., Posteraro B. (2017). Potential use of MALDI-TOF mass spectrometry for rapid detection of antifungal resistance in the human pathogen *Candida glabrata*. Sci. Rep..

[45] Paul S., Singh P., AS S., Rudramurthy S.M., Chakrabarti A., Ghosh A.K. (2018). Rapid detection of fluconazole resistance in *Candida tropicalis* by MALDI-TOF MS. Med. Mycol..

[46] Knoll M.A., Ulmer H., Lass-Flörl C. (2021). Rapid Antifungal Susceptibility Testing of Yeasts and Molds by MALDI-TOF MS: A Systematic Review and Meta-Analysis. J. Fungi.

[47] Paziani M.H., Carvalho L.T., Melhem M.D.S.C., de Almeida M.T.G., da Silva M.E.N.B., Martinez R., Santos C., Kress M.R.V.Z. (2020). First Comprehensive Report of Clinical *Fusarium* Strains Isolated in the State of Sao Paulo (Brazil) and Identified by MALDI-TOF MS and Molecular Biology. Microorganisms.

[48] Durand C., Maubon D., Cornet M., Wang Y., Aldebert D., Garnaud C. (2021). Can We Improve Antifungal Susceptibility Testing?. Front. Cell. Infect. Microbiol..

[49] Araújo E., Gusmão N., Silva T., Le Pape P., Lima-Neto R.G. (2022). MALDI-TOF MS-based evaluation for azole-susceptibility testing of Aspergillus fumigatus over reference broth microdilution method. Res. Sq..

[50] Faustino C., Pinheiro L. (2020). Lipid Systems for the Delivery of Amphotericin B in Antifungal Therapy. Pharmaceutics.

